# Time for implementation of local observational studies

**DOI:** 10.1097/j.pbj.0000000000000204

**Published:** 2023-04-10

**Authors:** Gonçalo M.C. Rodrigues, Maria J. Salgado, Ana P. Fernandes, Ana R. Jaime, Ana M. Macedo, Manuel Salavessa

**Affiliations:** a Departamento Médico – Hematologia, Janssen-Cilag, S.A., Lisboa, Portugal; b Departamento de Ciências Biomédicas e Medicina, Universidade do Algarve, Faro, Portugal

## To the Editor

The importance of improving clinical research is a consensual topic. Observational studies represent a practical approach to measuring real-world outcomes and responding to health policy directives by their descriptive nature and hypothesis generators. Thus, in the *era* of evidence-based medicine, the prompt approval of these studies would benefit their implementation and data collection.

This pilot study evaluated the approval times for eight observational studies in the fields of hematology, psychiatry, oncology, and immunology, in 43 Portuguese hospitals, between 2018 and 2021. Study objectives range from effectiveness, outcomes measurements, quality-of-life research, or patient pathway analyses. We considered 112 records corresponding to submissions to ethics committees (EC) and approval requests to administration boards. A descriptive analysis of the approval times was performed.

**Figure 1.**
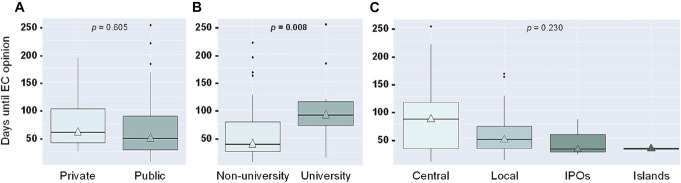
Time comparison until EC opinion between private *vs* public hospitals (A), non-university *vs* university hospitals (B), and central hospitals *vs* others (local, IPOs, and islands: Madeira and Azores; C).

The mean time to obtain EC authorization was 67 days (SD = 55; median = 46.5; 6–253), and the time for financial contracts' approval was 71 days (SD = 56; median = 53; 1–226). In 10% of the cases, the time to EC authorization was higher than 165 days, and financial contracts' approval surpassed 148 days. The mean time to final approval was 118 days (SD = 62; median = 99; 41–276). Among the considered records, 29% were approved in less than 30 days (6–29), 27% in less than 60 days (32–54), and 44% in more than 60 days (62–253). There were no differences between approval times in public *vs* private hospitals or central *vs* secondary hospitals. Still, university hospitals had a significantly longer median time for EC authorization (94 days *vs* 42 days in non-university hospitals, *P* = 0.008, calculated using the Mann-Whitney *U* test) (Fig. 1). While the increased approval times might either be a direct consequence of the hospitals' high internal flow or be due to the need to clarify submission-related issues raised by the hospitals, remarkably, the submissions approval rate is 100%.

A similar scenario was observed by Eichler et al, who analyzed the necessary time for institutional review boards to approve noninterventional studies in Germany and found that university hospitals needed more than three times the days that state medical chambers required (72.4 *vs* 20.3 days, respectively).^1^ Another analysis that described the differences in the processes to obtain EC approval for one study in 16 European/European-affiliated countries showed that time from application until first national approval ranged from less than a week (in Greece and the Netherlands) to more than 300 days (in Portugal).^2^

Although preliminary, these results suggest high heterogeneity among hospitals, highlighting the need to adopt improved strategies to standardize procedures across sites. Simultaneously, stakeholders involved in clinical studies (sponsors, clinical research organizations, investigators, and regulatory agencies) should implement preventive methods to meliorate EC authorization time and financial contracts' approval process. Study limitations include the small number of observations and the fact that some submissions coincided with the COVID-19 pandemic, which may have interfered with the usual procedures and times.

## Contributors

G.M.C.R. and M.J.S. were responsible for the conceptualization, investigation, data curation, and editing. A.R.J. was responsible for the conceptualization and editing. A.P.F. performed data curation and editing. A.M. was responsible for the conceptualization, methodology, and initial draft preparation. M.S. was responsible for the conceptualization, supervision, formal analysis, and review.

## Financial support and sponsorship

This research was sponsored by the pharmaceutical laboratory Janssen-Cilag.
